# Supplementary material for: Vitamins K2 and D3 Improve Long COVID, Fungal Translocation, and Inflammation: Randomized Controlled Trial
Source: Nutrients. 2025 Jan 16;17(2):304. doi: 10.3390/nu17020304 (PMC11767688; doi:10.3390/nu17020304)
Supplement: Supplementary file 1 [file nutrients-17-00304-s001.zip › nutrients-3424186-supplementary.pdf]

Vitamins K2 and D3 Improve Long COVID, fungal translocation, and Inflammation: Randomized controlled trial

Ornina Atieh

Supplementary material

Supplementary Table S1: Symptom Resolution by Type.

	**Baseline**				**6 months**				△		△ % change between treatment arms over study period (K2/D3 - SOC)
	D3/K2		SOC		D3/K2		SOC		D3/K2	SOC	
	n	%	n	%	n	%	n	%	%	%	
**Classification**											
⩾2 Symptoms	98	100.0	53	100.0	77	95.1	42	100.0	-4.9	0.0	-4.9
⩾ 12 LC Research Index	42	42.9	29	54.7	29	35.8	26	61.9	-7.1	7.2	-14.3*
CardioPulmonary Phentotype	81	82.7	49	92.5	63	77.8	34	80.8	-4.9	-11.7	6.8
Neurological Phenotype	88	89.8	49	92.5	70	86.4	36	85.7	-3.4	-6.8	3.4
General Phenotype	93	94.9	49	92.5	74	91.4	41	97.6	-3.5	5.1	-8.6
**Symptoms**											
Fatigue	67	68.4	41	77.4	47	58.0	28	66.7	-10.4	-10.7	0.4
Post Exertional Malaise	31	31.6	25	47.2	10	12.3	7	16.7	-19.3	-30.5	11.28*
Weakness in Arms	35	35.7	20	37.7	19	23.5	8	19.0	-12.2	-18.7	6.4
Fever, chills, sweats	22	22.5	15	28.3	8	9.9	5	11.9	-12.6	-16.4	3.8
Change in smell or taste	37	37.8	27	50.9	13	16.0	11	26.2	-21.8	-24.7	3.0
Pain in any part of the body	50	51.0	17	32.1	24	29.6	9	21.4	-21.4	-10.7	-10.7*
Shortness of breathe	45	45.9	29	54.7	24	29.6	19	45.2	-16.3	-9.5	-6.8
Persistent Cough	35	35.7	23	43.4	12	14.8	12	28.6	-20.9	-14.8	-6.1
Palpitations	30	30.6	20	37.7	14	17.3	11	26.2	-13.3	-11.5	-1.8
Swelling of legs	22	22.5	8	15.1	9	11.1	3	7.1	-11.4	-8.0	-3.4
Bladder problems	13	13.3	9	16.9	5	6.2	3	7.1	-7.1	-9.8	2.6

Nerve problems	28	28.6	14	26.4	10	12.3	4	9.5	-16.3	-16.9	0.6
Problems with anxiety	34	34.7	16	30.2	18	22.2	11	26.2	-12.5	-4.0	-8.5
Problems thinking	60	61.2	39	73.6	35	43.2	25	59.5	-18.0	-14.1	-3.9
Stopping breathing	19	19.4	16	30.2	7	8.6	8	19.0	-10.8	-11.2	0.4
Feeling faint or dizzy	36	36.7	25	47.2	15	18.5	14	33.3	-18.2	-13.9	-4.3
Changes in skin color	10	10.2	3	5.7	1	1.2	1	2.4	-9.0	-3.3	-5.6
Skin rash	11	11.2	8	15.1	1	1.2	4	9.5	-10.0	-5.6	-4.4
Excessively dry mouth	27	27.6	21	39.6	17	21.0	10	23.8	-6.6	-15.8	9.2
Excessive thirst	25	25.5	19	35.9	13	16.0	11	26.2	-9.5	-9.7	0.3
Vision problems	21	21.4	16	30.2	9	11.1	8	19.0	-10.3	-11.2	0.9
Problems with hearing	16	16.3	11	20.8	5	6.2	6	14.3	-10.1	-6.5	-3.6
Hair loss	26	26.5	14	26.4	11	13.6	7	16.7	-12.9	-9.7	-3.2
Problems with teeth	15	15.3	8	15.1	1	1.2	4	9.5	-14.1	-5.6	-8.5
Changes to menstrual cycle	14	14.3	8	15.1	3	3.7	0	0.0	-10.6	-15.1	4.5
Changes in fertility	1	1.0	4	7.6	0	0.0	0	0.0	-1.0	-7.6	6.6
Changes in sexual desire	15	15.3	9	16.9	5	6.2	2	4.8	-9.1	-12.1	3.0
Upper gastrointestinal distress	21	21.4	17	32.1	19	23.5	14	33.3	2.1	1.2	0.8
Lower gastrointestinal distress	23	23.5	20	37.7	24	29.6	14	33.3	6.1	-4.4	10.5*

**indicates a 10% difference between the vitamins D3/K2 and SOC arms*
Abbreviations: LC: Long COVID, SOC: Standard of care

Supplementary Table S2: Adverse Events Frequency by Type.

Adverse Event Type	Frequency
High Alkaline phosphatase (< 3x upper limit)	1
High ALT level (> 3x Upper limit)	3
High ALT level (< 3x Upper limit)	5
High AST level (> 3x Upper limit)	2
High AST level (< 3x Upper limit)	2
Borderline High total cholesterol (200 – 239 mg/dL)	40
High total cholesterol (240 mg/dL and above)	14
Elevated Creatinine level (1.17 – 2 mg/dL)	11
Low platelet level	2
Elevated Glucose levels (100 – 125 mg/dL)	29
Elevated Glucose levels (126 mg/dL and above)	14
Myocardial infarction	1
Above optimal LDL cholesterol level (100 – 129 mg/dL)	2
Borderline high LDL cholesterol level (130 – 159 mg/dL)	26
High LDL cholesterol level (160 – 189 mg/dL)	11
Low phosphorus levels	4
High Potassium level (> 6 mmol/L)	1
Low Sodium levels	2
High total bilirubin	2
Borderline high triglyceride levels (150 – 199 mg/dL)	16
High triglyceride levels (200 – 499 mg/dL)	15
Very high triglyceride level (500 mg/dL and above)	1
High Uric Acid level	8
Headache	1
**Total AE**	213

Abbreviations: ALT= Alanine aminotransferase; AST= Aspartate aminotransferase; LDL= Low-density lipoprotein.
